# Estimating Congestion in a Fixed-Route Bus by Using BLE Signals

**DOI:** 10.3390/s22030881

**Published:** 2022-01-24

**Authors:** Yuji Kanamitsu, Eigo Taya, Koki Tachibana, Yugo Nakamura, Yuki Matsuda, Hirohiko Suwa, Keiichi Yasumoto

**Affiliations:** 1 Graduate School of Science and Technology, Nara Institute of Science and Technology, Nara 630-0192, Japan; taya.eigo.td3@is.naist.jp (E.T.); tachibana.koki.ti0@is.naist.jp (K.T.); yukimat@is.naist.jp (Y.M.); h-suwa@is.naist.jp (H.S.); yasumoto@is.naist.jp (K.Y.); 2 RIKEN Center for Advanced Intelligence Project AIP, Tokyo 103-0027, Japan; 3 Graduate School and Faculty of Information Science and Electrical Engineering, Kyushu University, Fukuoka 819-0395, Japan; y-nakamura@ait.kyushu-u.ac.jp; 4 JST PRESTO, Tokyo 102-0076, Japan

**Keywords:** people counting, crowd density, BLE, route bus, machine learning

## Abstract

Information on congestion of buses, which are one of the major public transportation modes, can be very useful in light of the current COVID-19 pandemic. Because it is unrealistic to manually monitor the number of riders on all buses in operation, a system that can automatically monitor congestion is necessary. The main goal of this paper’s work is to automatically estimate the congestion level on a bus route with acceptable performance. For practical operation, it is necessary to design a system that does not infringe on the privacy of passengers and ensures the safety of passengers and the installation sites. In this paper, we propose a congestion estimation system that protects passengers’ privacy and reduces the installation cost by using Bluetooth low-energy (BLE) signals as sensing data. The proposed system consists of (1) a sensing mechanism that acquires BLE signals emitted from passengers’ mobile terminals in the bus and (2) a mechanism that estimates the degree of congestion in the bus from the data obtained by the sensing mechanism. To evaluate the effectiveness of the proposed system, we conducted a data collection experiment on an actual bus route in cooperation with Nara Kotsu Co., Ltd. The results showed that the proposed system could estimate the number of passengers with a mean absolute error of 2.49 passengers (error rate of 38.8%).

## 1. Introduction

Buses are one of the main forms of public transportation. Information on crowding, or congestion, on buses can be very useful in light of the current COVID-19 pandemic for both users and society in terms of avoiding congestion for individuals and equalizing congestion in society. We believe that bus operators can improve their business by having information on bus congestion, which can help them adjust the number of buses, change their operating hours, and optimize their routes. In addition, we believe that passengers can use buses in a systematic way to reduce the risk of infection, for example, by riding buses that are less crowded, as determined from information about congestion on the bus. Thus, there is a need for a service that visualizes real-time bus congestion information, a route recommendation service that considers the degree of crowding to minimize the risk of infection, and a service that predicts the degree of crowding in the future.

To provide these services, it is necessary to obtain congestion information for a bus in advance. Because it is unrealistic to manually obtain congestion information for all currently operating buses, a system that can automatically obtain such information is necessary. It is possible to obtain the number of bus users on particular routes using information from prepaid transportation cards. However, many suburban areas and sparsely populated areas have not adopted such cards, and using them for ridership information has problems such as not being able to include commuter pass users and users who pay with cash. Hsu et al. proposed a method to estimate the number of passengers using a deep learning algorithm based on video data obtained from two cameras installed in a bus [1]. This method shows a high accuracy of about 2.0 MAE on a crowded dataset. However, due to the specification of using two cameras, the cost of installation and the privacy of passengers are not taken into account, and it is not easy to implement the method in actual operation. Yamada et al. have developed a system for detecting passengers using a LiDAR sensor that outputs distance data to objects [2]. An algorithm that reduces the amount of computation enables highly accurate estimation with an inexpensive single-board computer. However, the system is vulnerable to overlapping passengers, so the accuracy is low in extremely crowded conditions. Additionally, the LiDAR sensor is very expensive and there are restrictions on the location of the device. To implement the system on an actual bus, it is necessary to design a system that satisfies two main requirements: the collection of data that does not include private passenger information and a low installation cost for the sensing devices.

Recently, with the rapid spread of smartphones, wireless communication technologies such as WiFi and BlueTooth are receiving more attention in crowd behavior and crowd density estimation [3]. Abedi et al. presents the benefits and critical challenges of the use of Bluetooth and WiFi for crowd data collection and monitoring [4]. In this paper, we propose a congestion estimation system that protects passengers’ privacy and reduces installation costs by using Bluetooth low-energy (BLE) signals as sensing data. The proposed system consists of (1) a sensing mechanism that acquires BLE signals emitted from bus passengers’ mobile terminals and (2) an estimation mechanism that determines the degree of congestion in the bus from this data. The sensing mechanism obtains the Bluetooth device (BD) address to identify the device and also obtains the received signal strength indicator (RSSI) included in the BLE signal. Devices change their BD address at regular time intervals for privacy reasons, so there is little risk of privacy violation. In addition, BLE signals can be received by a single inexpensive and lightweight single-board computer, such as a Raspberry Pi, without the use of special sensors, and there are no restrictions on the installation location. Furthermore, if a device capable of receiving BLE signals is already present on a bus, the system can be implemented by simply installing software. The estimation mechanism uses the data obtained by the sensing mechanism to estimate the degree of congestion on a bus route. Two methods of estimation are considered. One is to set a threshold value for the RSSI for the obtained BD addresses and use the total number of addresses that satisfy the threshold value as valid addresses. The other is to use a machine learning model. In the machine learning method, the total number of addresses is dealt with as one of the feature values. The model is trained by constructing a dataset that includes information specific to the bus route, such as the operating time and route number. We also compare the performance of the model with that of a model trained on a dataset that does not contain such information.

To evaluate the effectiveness of the proposed system, we conducted a data collection experiment on an actual bus route with Nara Kotsu Co., Ltd. (Nara, Japan, https://www.narakotsu.co.jp/ (accessed on 9 December 2021)) The data collected covered a wide range of time periods, from the morning commute to the evening return home, from the central area of Nara City to the sparsely populated areas in the south, and from children to elderly passengers. The types of data included BD address, the RSSI included in the BLE signal, location information, and time information. In the results for estimating the number of passengers based on the data obtained in the experiment, the estimation by threshold showed a mean absolute error (MAE) of 3.4 passengers (error rate 61.4%), and the estimation by machine learning showed an MAE of 2.49 passengers (error rate 38.8%). We confirmed that the accuracy was greatly improved by adding information specific to the bus route.

The contributions of this article include the following:

- We propose a BLE-based congestion estimation system that protects the privacy of passengers while reducing the cost of installation.
- We propose new features of learning models for estimating congestion of public route buses.
- Our estimation model, which is trained data from real-world experiments, achieves high accuracy for practical use.

## 2. Related Work

There are many studies on detecting people and estimating human congestion [5,6,7,8,9,10,11,12,13]. In this section, we introduce a study on estimating the number of passengers, and a study on congestion estimation using BLE signals which are particularly relevant to our study.

### 2.1. Estimating the Number of Passengers

Methods using a camera for estimating the number of passengers have been proposed [14,15,16]. Song et al. proposed a system for counting passersby using video data obtained from surveillance cameras [14]. Although such a camera-based system provides a more accurate estimation of the number of passengers, it is not easy to implement in local buses because of the high installation and operation costs of the system and the fear of privacy violations.

Methods using an infrared sensor for detecting the passengers have also been proposed [17,18,19,20,21]. In order to measure the number of passengers in airports, Bauer et al. proposed the system combining an infrared sensor and mat-type pressure sensor. This method uses a small infrared sensor which can be easily installed in various locations. However, the detection range of the infrared sensor is limited, so multiple sensors are required, which increases the installation cost.

In addition, Methods for estimating crowd density using WiFi signals have also been proposed [22,23]. Handte et al. used WiFi signals emitted from passengers’ mobile devices to estimate the number of passengers on a bus route [22]. The error rate was 5.1 people, but it increased when the bus was crowded. In addition, the estimation using WiFi risks violating privacy because it obtains a unique MAC address for each device. Hidayat et al. proposed a new data cleaning procedure to characterize bus passenger volume using a combination of media access control (MAC) address and global positioning system (GPS) data for estimating the number of passengers [24,25]. Their approach shows that the correlation between WiFi estimation and ground truth is 0.78.

### 2.2. Congestion Estimation Using BLE Signals

With the widespread use of smartphones, many studies on estimation of crowd density using wireless communication technologies such as BLE have studied [26,27,28,29,30]. Table 1 shows a comparison of our proposed method and these studies.

Umeki et al. focused on the RSSI intensity, which varies greatly depending on the number of people, and proposed a system to estimate the congestion level of a sightseeing spot in real time by observing the RSSI intensity distribution using BLE devices installed at the spot [28]. While it is possible to estimate the degree of congestion at tourist spots without requiring tourists to wear special equipment, it is necessary to install two BLE devices, a transmitter and a receiver, in the environment. This method estimates the degree of congestion in three levels: low, medium, and high.

Weppner et al. proposed a method to estimate the crowd density by aggregating the number of nearby BLE terminals detected from the mobile signals of users moving in the environment [29,31]. Their method could effectively fuse information obtained from multiple terminals through user-participatory sensing, and estimate the degree of congestion without the need to install sensors in advance. However, the estimation accuracy strongly depended on the proportion of users participating in the sensing.

These methods using wireless communication technologies such as BLE take privacy into account. However, in actual operation, it is necessary to consider not only the privacy of passengers but also the installation cost of the device (number of sensors required, location constraints).

### 2.3. Positioning of This Research

This paper proposes a system for estimating the degree of congestion on a bus route that protects the privacy of passengers and reduces the installation and operation cost of the system. To evaluate the effectiveness of the system, we conducted a data collection experiment on actual bus routes in Nara Prefecture. Although there have been studies on detecting passengers in buses, they have been conducted only under limited environments and conditions, and few studies have considered actual operation. In addition, it is a unique approach to estimate the level of congestion on a bus route, whose usage varies greatly depending on the region and time of day, by accounting for information such as the operating time and route number. This system does not require any special sensors and can be operated using a single-board computer, such as a Raspberry Pi, which is inexpensive and lightweight. In addition, there are no restrictions on the location of the system, and the system is designed with privacy in mind and to reduce operating costs.

## 3. Proposed System

In this section, we summarize the system requirements and then propose a system that satisfies the requirements.

### 3.1. System Requirements

The requirements of the system to be implemented on an actual bus route are as follows:

- Collection of data that does not include passenger privacy information
- Reduction of the installation cost of sensing devices

One of the major barriers to introducing a system into actual buses is the issue of passenger privacy. To solve this problem, it is necessary to collect data that do not contain private passenger information. A camera-based estimation method can provide more accurate estimation, but it also obtains private information, namely face images. As a solution, we can reduce the risk of privacy violation by not uploading the privacy data to a server, and instead discarding it immediately after the estimation is done locally. However, it is necessary to install the system at a location where it can see the entire bus, such as on the ceiling, which increases the installation and operation costs. Therefore, to ensure the privacy of passengers and make the device location flexible, it is necessary to collect data that does not include private information and make the device small and compact. In addition, it is necessary to design a system that does not place restrictions on the location of the devices.

### 3.2. Overview of the Proposed System

**Figure 1:**
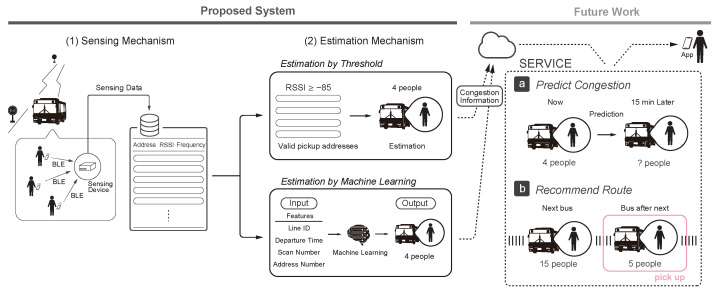
Schematic diagram of the proposed system.

Based on the system requirements in Section 3.1, we propose a system for estimating the congestion level on a bus route considering the actual operation. The outline of the proposed system is shown in Figure 1. The proposed system consists of (1) a sensing mechanism to collect data for a bus and (2) an estimation mechanism to estimate the degree of congestion from the data obtained by the sensing mechanism. These are explained in detail in Section 3.3. The figure also shows the use cases of the system: (a) future congestion prediction service and (b) congestion-aware route recommendation service. In the case of (a), the future congestion prediction service will provide users with information on the future congestion level on the bus routes. This will enable passengers to use the bus avoiding congestion. In addition, we believe that in the case of (b), the congestion-aware route recommendation service can save users the trouble of route selection by recommending routes that take into account the level of congestion as well as the cost of travel fees and travel time.

### 3.3. System Design

(1) Sensing Mechanism

The sensing mechanism uses BLE signals emitted from the passengers’ mobile terminals as sensing data. BLE is a power-saving communication standard among the short-range wireless communication standards called Bluetooth. To connect with other BLE devices, a BLE device continuously broadcasts a communication signal, which is a one-way transmission of data to an unspecified number of parties. The data transmitted includes the BD address to identify the device and an RSSI to indicate the signal strength. The sensing mechanism obtains the BD address and RSSI from the data contained in the BLE signal emitted from the passengers’ mobile terminal. Because the BD address is randomly changed at certain time intervals for privacy reasons, there is little risk of violating the passengers’ privacy. In addition, BLE enables sensing without the use of special sensors, and if the bus already has a device that can receive BLE signals, the system can be operated by simply installing software without the need to install a sensing device.

(2) Estimation Mechanism

In the estimation mechanism, the degree of crowding on a bus is estimated from the data obtained by the sensing mechanism. In this study, we estimated the number of passengers between stops as an indicator of bus congestion. We consider the possibility of detecting addresses outside the bus. In light of this problem, we cannot simply associate the number of BD addresses detected with the number of passengers. Therefore we first propose a threshold-based estimation that sets a threshold value for the RSSI of BD addresses obtained by the sensing mechanism, and the total number of addresses that equals or exceeds the threshold is used as the estimated value. On the other hand, we assumed that some passengers on the bus do not have a mobile terminal, while others have multiple mobile terminals. In addition, as mentioned in the previous section, the BD address changes after a certain period of time, so there is a possibility that the same terminal is counted twice. In order to deal with these problems, we next propose a machine learning-based estimation that uses the total number of addresses and information specific to the bus route as features.

## 4. Implementing the Data Collection Device

In this section, we describe the sensing device for data collection implemented in this study.

### 4.1. Implementation Overview

To evaluate the effectiveness of the proposed system, we implemented a sensing device for data collection in the data collection experiment described in Section 5.

### 4.2. Sensing Device

**Figure 2:**
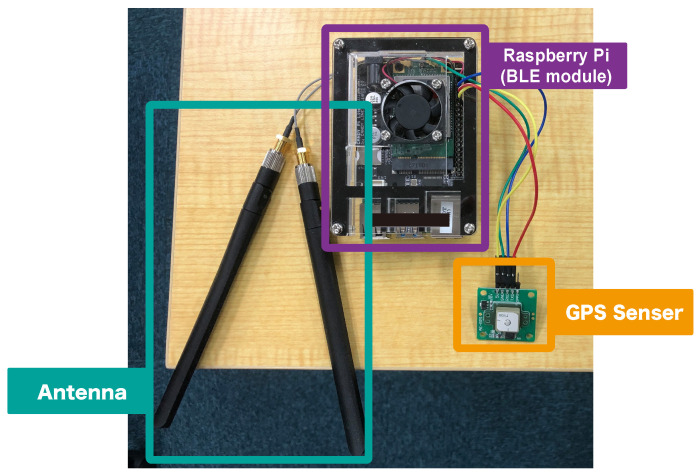
The sensing device.

The implemented device is shown in Figure 2. A Raspberry Pi 4 (https://www.raspberrypi.org/products/raspberry-pi-4-model-b/ (accessed on 9 December 2021)) capable of BLE communication was used as the sensing device. To acquire positional information, we installed a GPS module (https://akizukidenshi.com/catalog/g/gK-09991/ (accessed on 9 December 2021)) on the Raspberry Pi. To remotely check system operation, the acquired data were sent to a server. Because the bus did not have an internet connection, we installed a communication module (https://candy-line.com/portfolio/candy-pi-lite-lte-m/ (accessed on 9 December 2021)) on the Raspberry Pi. Because these modules could be incorporated into the single-board Raspberry Pi, it was possible to reduce the overall size of the sensing device. The data that can be acquired using the sensing device includes the BD address and RSSI contained in the BLE signal, as well as the location and time during data acquisition.

### 4.3. Sensing Process

**Figure 3:**
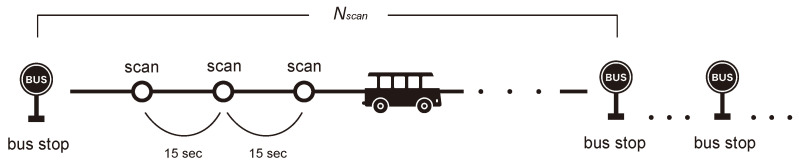
Sensing process.

In this study, we estimated the number of passengers between bus stops as an indicator of the degree of congestion on the bus route. The information of bus stops is given from Nara Kotsu Co., Ltd. Therefore, we know the exact location of bus stops. The bus stops are identified using the location information obtained from GPS. The sensing process is shown in Figure 3.

This system detects the surrounding BLE terminals once every 15 s. On some routes, the time interval between stops (the time it takes from leaving one stop to halting at the next stop) is less than 1 min. Here, the total number of detections between stops is $N_{scan}$. The BD address and RSSI of the detected BLE terminals are stored as data. We assume that $N_{scan}$ includes multiple detections of the mobile terminals of bus passengers. The number of times the same BD address is detected is denoted as $n_{detected}$. The RSSI of the *i*th detection is S(i), and the mean value of RSSI $S_{mean}$ and the frequency of occurrence *F* (%) are defined by Equations (1) and (2), respectively.

(1) $$\;S_{mean}=\frac{1}{n_{detected}}\sum_{i=1}^{n_{detected}}S\left(i\right),$$

(2) $$\;F\left(\%\right)=\frac{n_{detected}}{N_{scan}}\times 100.$$

An example of the sensing data obtained between stops is shown in Table 2.

## 5. Data Collection Experiment

### 5.1. Experiment Overview

**Figure 4:**
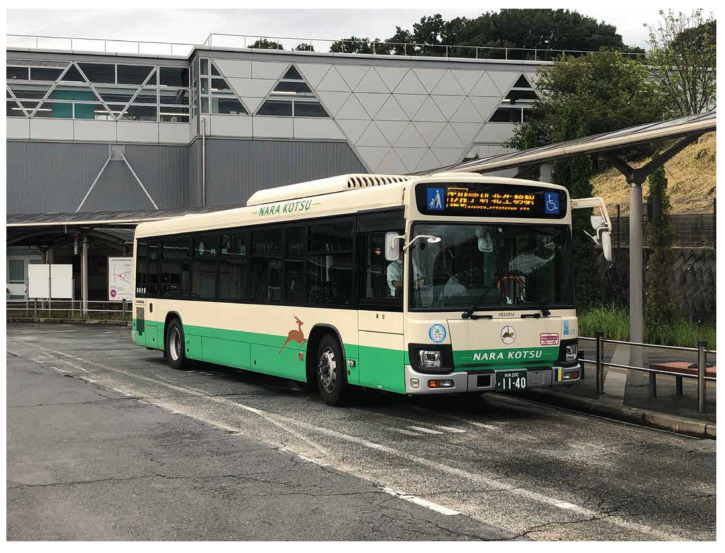
The actual bus in cooperation with Nara Kotsu Co., Ltd.

**Figure 5:**
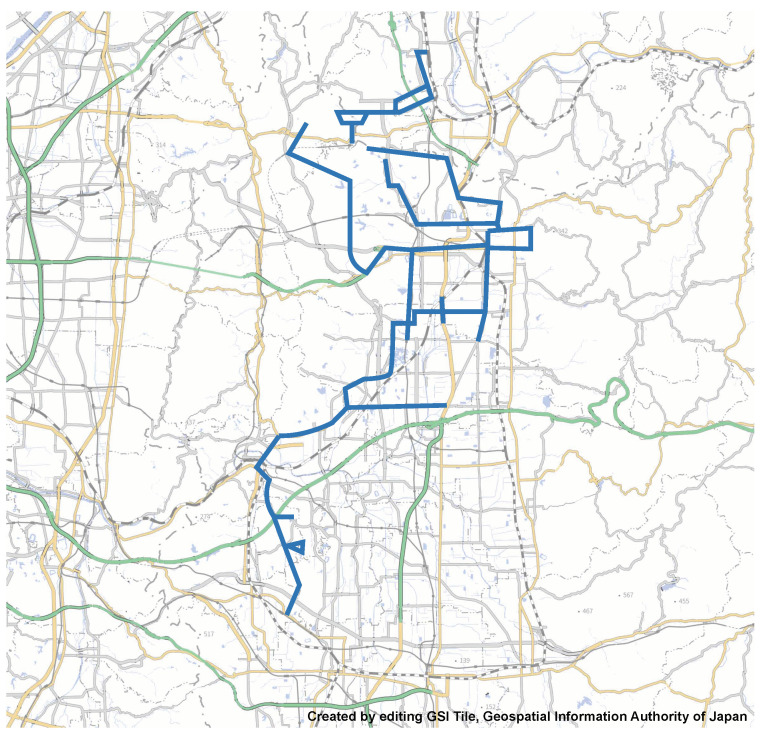
The data collection area.

To evaluate the effectiveness of the proposed method, a data collection experiment was conducted on 21 December 2020, using actual buses in cooperation with Nara Kotsu Co., Ltd. (Figure 4). The bus seats about 20 people and has a maximum capacity of about 40 people. The data were collected from 39 routes (with overlaps) in Nara Prefecture (shown as a blue line in Figure 5). The number of passengers on a bus route varies by the time of day, such as rush hours for commuting or returning home. Therefore, we conducted an experiment between 7:00 a.m. and 7:00 p.m. to collect data at various times. To conduct the experiment on an actual bus route, the following three restrictions were imposed: (1) no power supply from the bus, (2) no sensing device can be installed, and (3) the actual number of passengers cannot be given. Therefore, the experimenter brought the sensing device along with a mobile battery onto the bus to collect data and visually check the number of passengers in the bus to obtain the actual passenger counts. For this purpose, the experimenter sat at the rear of the bus, where it was easier to observe the entire interior. Therefore, the sensing device was located at the same position as the experimenter.

### 5.2. Results of the Experiment

We obtained a total of 662 stop-to-stop datasets for 39 routes. Each inter-stop dataset included the departure time from the stop, the total number of BD addresses, and the actual number of passengers. The results of the route section between Gakken Nara Tomigaoka Station and Takanohara Station are shown in Table 3 as a part of the experimental results from the 39 routes.

**Figure 6:**
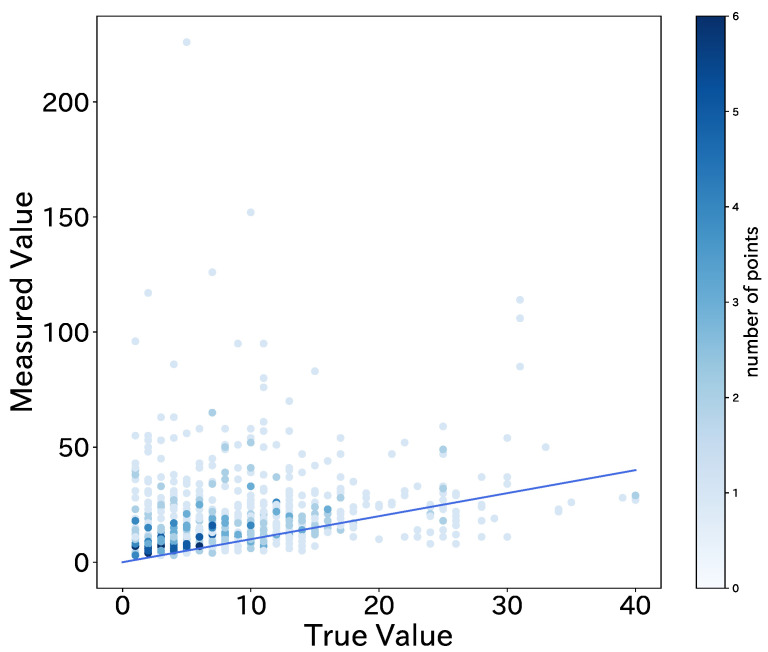
Measured values (BD address counts) versus true values (passenger counts) obtained from raw data.

A graph plotting the total number of BD addresses (measured value) on the vertical axis and the number of passengers (true value) on the horizontal axis for the 662 raw datasets is shown in Figure 6.

The blue line in the graph (y=x) represents the ideal state of no error between the true value and the estimated value. As can be seen from this graph, the total number of BD addresses obtained between each stop and the actual number of passengers are far apart. This can be attributed to the fact that, in addition to the mobile terminals owned by the passengers, signals are also received from people outside the bus and other BLE terminals in the city. In addition, many passengers have multiple BLE devices, so it is difficult to simply use the total number of addresses obtained as the number of passengers. Therefore, in this study, threshold estimation was used to determine whether the obtained BD addresses are valid by setting thresholds for the RSSI and frequency of occurrence and machine learning models.

## 6. Estimation and Evaluation

In this section, we describe the estimation of the number of passengers on a bus using the proposed estimation mechanisms identified as (2) in Section 3.3.

### 6.1. Estimation by Threshold

As can be seen from Section 5.2, it is difficult to estimate the number of passengers on a bus route simply from the total number of BD addresses in the sensing data. To solve this problem, we set thresholds for the average RSSI and frequency of occurrence of BD addresses to determine the validity of addresses, and then used the total number of valid addresses as the number of passengers. We assume that the signal of the device in the bus indicates the stronger RSSI and the higher frequency of occurrence. In order to evaluate the proposed method, the score that shows the highest accuracy when a threshold is set only for RSSI is adopted as the baseline in this study.

**Figure 7:**
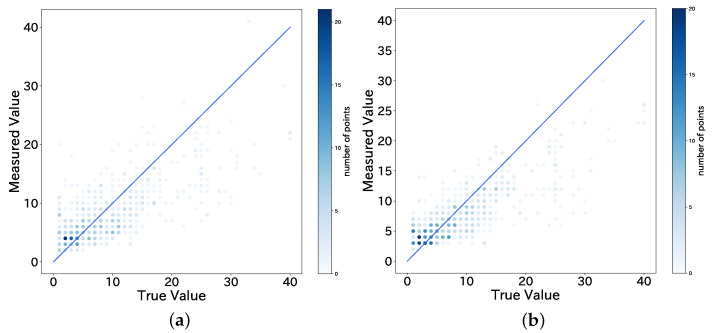
Values estimated by threshold (number of BD addresses) versus true values (number of passengers). (**a**) Baseline (RSSI ≥ −74); (**b**) Proposed (RSSI ≥ −80, F ≥ 40%).

The MAE and the mean absolute percentage error (MAPE) averaged over all the stops are calculated, and the thresholds of the mean RSSI and frequency are chosen to achieve the smallest MAE. The results are shown in Table 4. The threshold estimation method performs poorly because it cannot deal with the case in which the observed value is lower than the true value. As can be seen from Figure 7, the threshold estimation method performs poorly when the estimated value is lower than the true value.

### 6.2. Estimation by Machine Learning Models

As mentioned in Section 3.3 (2), there are some remaining problems, such as passengers who have multiple terminals or no terminals. Therefore, instead of using the total number of addresses as the number of passengers, we propose a machine learning estimation method that deals with the total number of addresses as a feature and takes into account information such as the operating time of the bus route and the route number. It is believed that data such as departure times and route numbers contain important information for estimating the degree of congestion because they indicate conditions such as commuter rush, going home rush, urban area, and suburban area. Thus, we proposed a machine learning model that accounts for information specific to bus routes. For each of the 662 inter-stop datasets, 19 feature dimensions are given. We believe that the RSSI and frequency of occurrence of a BD address are important indicators to evaluate the effectiveness of the address. Therefore, we added the total number of each address to the feature when the threshold is set in steps. This gave us 16 features for the number of addresses. In addition to these, three other features specific to route buses are route ID, departure time, and the total number of detections. To compare the results of the different methods, we constructed two types of datasets: a dataset called the Narabus dataset (ND) which does not include the three bus-specific features and another dataset, called ${\mathrm{ND}}^{+}$, which includes all the features.

**Figure 8:**
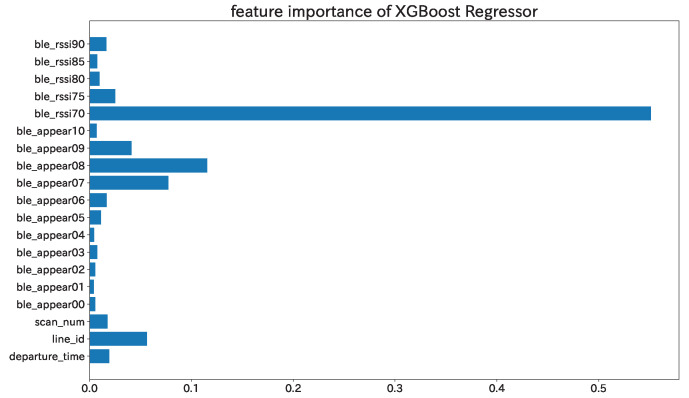
The feature importance of the XGB model.

Four types of learning models, linear regression (LR), a support vector machine (SVM), a random forest (RF), and XGBoost (XGB), were employed. In regards to the evaluation, we first divide all 662 data into a 3(496):1(166) ratio of training data and test data. Then, we run a grid search using leave-one-out cross-validation and train models with train data. Finally, we calculate the performance of each model with test data. As in the threshold estimation, MAE and MAPE were calculated as evaluation indices, and the results are shown in Table 5. For all the machine learning models, we confirmed that the performance was improved by using the dataset ${\mathrm{ND}}^{+}$, which contains features specific to bus routes, as input. Among the models, XGB showed the best accuracy, with an error rate of 38.8%. The feature importance of the XGB model is shown in Figure 8. “ble_rssixx” and “ble_appearyy” mean the total number of addresses with average RSSI xx or higher and the total number of addresses with the frequency of occurrence yy or higher, respectively.

**Figure 9:**
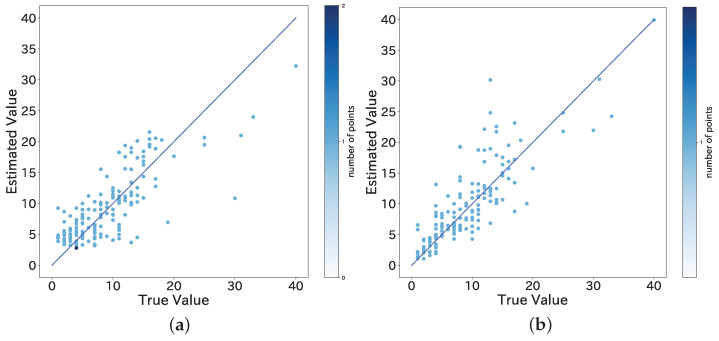
Values estimated by machine learning versus true values (number of passengers). (**a**) XGB (ND); (**b**) XGB (${\mathrm{ND}}^{+}$).

A graph plotting the measured value on the vertical axis and the true value on the horizontal axis is shown in Figure 9.

## 7. Discussion

Looking at the raw data of Figure 6, we can see that when the true value is less than 20 people, we obtain more addresses than the true value for most of the inter-stop data. One of the possible causes is the reception of signals from BLE terminals outside the bus, as described in Section 5.2. To deal with this problem, we set appropriate thresholds for the average RSSI and frequency of occurrence of BD addresses and selected only valid addresses, which greatly reduced the error, as seen in Table 4. Ji et al. reports on the attenuation of the BLE signal with distance [32]. According to the report, it can be confirmed that the signal is attenuated as the distance increases. On the other hand, variations in the attenuation of the signal are shown even at the same distance. The variability of the signal attenuation may have a significant impact on the threshold estimation and machine learning estimation in this study. As a future work, we will consider a method to set a dynamic threshold for each section, for example, in order to make the collection robust to signal noise. In addition, from Figure 8, it was confirmed that BLE signals with high signal strength (−70 or higher) are effective for counting the number of passengers. The signals with weak strength are thought to be acquired from devices outside the bus and are likely to be noise. In the future, it will be possible to acquire signals more efficiently by setting a suitable threshold value.

In contrast, when the true value of the raw data was more than 20 people, the number of detected addresses tended to be less than the true value. This may be due to BLE signals being blocked by people and objects in the crowded bus, making it difficult for BLE signals to propagate and be detected properly. Ma et al. show that the human body can attenuate BLE signal [33]. In addition, because the sensing device was located at the rear of the bus due to the limitations of the experiment, we believe that it could not detect the BLE devices of some of the passengers in front of it. One possible solution is to install the sensing device in the center of the bus or to use two devices, one in the front and one in the rear, but this would impose restrictions on the installation position of the devices and increase the cost of installation, which is a dilemma.

In this study, to solve the aforementioned problem, we used the machine learning model with added information specific to the bus route, such as time and route information, to improve the accuracy. The results of the performance evaluation showed that the model with the best accuracy had an error rate of 38.8%, which is a significant improvement over the estimation using a threshold. Figure 9 also shows a decrease in the error when the true value is more than 20 people. In addition, to examine whether the estimation is sufficient in congested situations, we re-calculated the MAE and the MAPE using only data with a true value of 10 or more people. The results showed that for the dataset ${\mathrm{ND}}^{+}$, the XGB model had an MAE of 3.7 people and a MAPE of 25.7%. This result confirms that the estimation can be done under the condition of crowded buses without any loss of accuracy. Three of the machine learning models (SVM, RF, XGB) selected for this study are known for their accuracy, and Table 5 confirms that the accuracy of these models is significantly higher than that of the most lightweight linear regression model. However, these models are not very lightweight compared to simple linear regression models. It is important to reduce the weight of the models in order to perform real-time estimation in the automation of congestion estimation, and this is an issue that needs to be addressed in the future.

## 8. Conclusions

In this study, we proposed a system for estimating the degree of congestion on a bus route using BLE signals as sensing data to protect passengers’ privacy and reduce installation costs, taking into account that the system will be installed on a bus that is actually in operation. To count passengers correctly who have multiple BLE devices and passengers who do not have any devices, we developed a machine learning model that accounts for information specific to bus routes, such as passenger demographics, that vary by region. This system consists of a single-board Raspberry Pi computer, which is inexpensive and lightweight. The system can be implemented by only installing software if the bus already has a device that can receive BLE signals. This has the advantage of installation without any restrictions on the location. As a result of evaluating the effectiveness of the proposed system by conducting data collection experiments on an actual bus, we showed that the system can estimate the number of passengers with high accuracy enough for practical use.

One of the future challenges is to design a system that can accurately acquire the BLE terminals of passengers even under crowded conditions. In consideration of actual operation, it is desirable to use a method that does not impose restrictions on the installation position of the sensing device. One possible solution is a participatory sensing method that uses the smartphones carried by passengers as sensing devices. In addition, we are planning to conduct a data collection experiment using a new actual bus route, and we will attempt to improve the accuracy by building a new model from the obtained data. In the future, we are going to monitor the level of congestion in all public transportation systems, and aim to solve social problems such as avoiding congestion for individuals and equalizing congestion in society.

## Figures and Tables

**Table 1:** Comparison table between related work and proposed method.

	Domain	Subject	Sensor	Privacy	Number of Sensors	Location Constraints	Estimate
[26]	indoor	pedestrian flow	BLE	◯	1	△	correlation
[27]	outdoor	pedestrian flow	Wi-Fi	△	3	△	correlation
[28]	outdoor	congestion	BLE	◯	2	△	classification
[29]	outdoor	congestion	BLE	◯	2	△	classification
[30]	bus	onboard devices	BLE	◯	1	△	correlation
Proposed	bus	number of passengers	BLE	◯	1	◯	regression

**Table 2:** Example of sensing data acquired between stops.

BD Address	The Mean Value of RSSI	The Frequency of Occurrence
00:00:5e:00:53:1a	−78.5	25
00:00:5e:00:53:38	−90.0	100
00:00:5e:00:53:90	−56.4	75
…	…	…

**Table 3:** A part of the experimental results (Gakken Nara Tomigaoka Station–Takanohara Station).

Departure Time	Bus Stop	Number1 ${}^{*a}$	Number2 ${}^{*b}$
08:52	Gakken Nara Tomigaoka	25	4
08:54	Kita Tomigaoka Ittyoume	25	4
08:55	Higashi Tomigaoka Yontyoume	25	5
08:56	Higashi Tomigaoka Gotyoume	25	6
08:57	Higashi Tomigaoka Rokutyoume	51	7
08:58	Tomigaoka Rokutyoume Higashi	104	8
09:00	Oshikuma/Jinkou	75	11
09:02	Seika Sakuragaoka Santyoume	40	15
09:03	Kabutodai Santyoume	44	15
09:04	Kabutodai Nityoume	51	15
09:05	Kabutodai Ittyoume Nishi	21	15
09:05	Kabutodai Ittyoume	78	15

${}^{*a}$ Number1 means that the total number of BD address of the sensing data; ${}^{*b}$ Number2 means that the total number of passengers in the bus (true value).

**Table 4:** Results of threshold estimation.

Method	MAE	MAPE
All	75.8	2182.5
Baseline (RSSI ≥ −74)	3.9	77.3
Proposed (RSSI ≥ −80, F ≥ 40%)	3.4	61.4

**Table 5:** Performance of each model for each dataset.

	ND		${\mathrm{ND}}^{+}$	
**Model**	**MAE**	**MAPE**	**MAE**	**MAPE**
LR	3.65	81.4	3.41	64.4
SVM	3.09	66.5	2.97	44.7
RF	2.93	63.1	2.54	47.7
XGB	2.98	60.0	2.49	38.8

## Data Availability

The data presented in this study are available on request from the corresponding author.
